# The impact of adjuvant surgical treatment of nontuberculous mycobacterial pulmonary disease on prognosis and outcome

**DOI:** 10.1186/s12931-020-01420-1

**Published:** 2020-06-16

**Authors:** Kiyoharu Fukushima, Mari Miki, Yuki Matsumoto, Emi Uda, Yuji Yamamoto, Yuya Kogita, Yuko Kagawa, Takanori Matsuki, Hiroyuki Kagawa, Yohei Oshitani, Daisuke Motooka, Kazuyuki Tsujino, Kenji Yoshimura, Keisuke Miki, Akio Hayashi, Shota Nakamura, Seigo Kitada, Yukiyasu Takeuchi, Hiroshi Kida

**Affiliations:** 1 Department of Respiratory Medicine, National Hospital Organization Osaka Toneyama Medical Center, 5-1-1 Toneyama Toyonaka, Osaka, Japan; 2 Department of Respiratory Medicine and Clinical Immunology, Osaka University Graduate School of Medicine, 2-2 Yamadaoka, Suita, Osaka, Japan; 3 Department of Infection Metagenomics, Genome Information Research Center, Research Institute for Microbial Diseases (RIMD), Osaka University, 3-1 Yamadaoka, Suita, Osaka, Japan; 4 Department of General Thoracic Surgery, National Hospital Organization Osaka Toneyama Medical Center, 5-1-1 Toneyama Toyonaka, Osaka, Japan; 5 Department of Respiratory Medicine, Yao Tokusyuukai General Hospital, 1-17 Wakakusa-cho, Yao, Osaka, Japan

**Keywords:** Nontuberculous mycobacteria, *Mycobacterium avium*, *Mycobacterium abscessus*, Thoracic surgery

## Abstract

**Background:**

Lung resection in patients with nontuberculous mycobacterial pulmonary disease (NTM-PD) has been reported to be associated with favorable outcomes. However, little is known regarding the risk and prognostic factors for refractory and recurrent cases. We aimed to evaluate the overall impact and benefit of adjuvant lung surgery by comparing NTM-PD patients who underwent adjuvant lung resection with those treated exclusively with antibiotics. We also investigated the efficacy of serum IgA antibody against glycopeptidolipid (GPL) core antigen (GPL core antibody) to monitor disease activity and predict the recurrence of disease after adjuvant lung resection.

**Methods:**

We retrospectively evaluated the clinical characteristics and surgical outcomes of 35 patients surgically treated for NTM-PD. Furthermore, we compared surgically treated patients and control patients treated exclusively with antibiotics who were matched statistically 1:1 using a propensity score calculated from age, sex, body mass index, and radiologic features of disease.

**Results:**

In the surgically treated patients, the median age was 58 (interquartile range, 47–65) years and 65.7% were female. Twenty-eight patients had *Mycobacterium avium* complex. Operations comprised four pneumonectomies, two bilobectomies, one bilobectomy plus segmentectomy, 17 lobectomies, two segmentectomies, and nine lobectomies plus segmentectomies. Postoperative complications occurred in seven patients (20%), there were no operative deaths, and 33 (94.3%) patients achieved negative sputum culture conversion. Refractory and recurrent cases were associated with remnant bronchiectasis, contralateral shadows, and positive acid-fast bacilli staining or culture. Of 28 statistically matched pairs, long-term sustained negative culture conversion was observed in 23 (82.2%) surgical group patients and in 14 (50.0%) non-surgical group patients (0.0438). The mortality rate was lower in the surgical group, but did not reach statistical significance (one in the surgical group and four in the non-surgical group, *p =* 0.3516). GPL core antibody was correlated with disease activity and recurrence.

**Conclusions:**

NTM-PD patients who underwent adjuvant lung resection experienced overall favorable outcomes and achieved sputum culture conversion more frequently. Long-term mortality may have been reduced by this procedure, and the level of GPL core antibody was shown to be a good clinical indicator of disease activity after surgery.

## Background

Non-tuberculous mycobacterial pulmonary disease (NTM-PD) has been increasingly implicated in a broad range of infectious diseases worldwide [1, 2]. Proper diagnosis and management of this disease are extremely important. In Japan, the most common cause of NTM-PD is the *Mycobacterium avium* complex (MAC), followed by *M. kansasii* and *M. abscessus* complexes [1]. With regard to the treatment of NTM-PD, although multiple antimicrobial regimens including macrolides have advanced in the last decade [3], the success rates of combination antibiotic treatment are unsatisfactory and microbiological recurrence even after successful completion of antibiotic therapy is relatively common [4–7]. Thus, as a chronic lung disease, NTM-PD exists as a significant health burden on various populations and is an important cause of morbidity and mortality [8, 9]. Controversies exist in the use of pulmonary resection in combination with multiple antimicrobial therapy; some studies reported favorable outcomes in terms of conversion and postoperative morbidity and mortality, but others did not, particularly for pneumonectomy [10–16]. Furthermore, little is known regarding the prognostic factors after long-term follow-up of patients with NTM-PD treated by pulmonary resection, and reports on the impact of remnant lesions following pulmonary resection on treatment failure, which includes clinical recurrence, have been conflicting [15, 16]. To prevent the development of respiratory failure, adjuvant lung resection to control bacterial burden in selected patients with refractory NTM-PD who can tolerate the procedure is a powerful option that can prevent disease progression [11, 12]. Therefore, it is important to clarify the indications for surgery and outcomes among NTM-PD patients who have undergone adjuvant lung resection. In this study we aimed to: 1) evaluate the clinical outcomes of pulmonary resection for NTM-PD; 2) determine the prognostic factors after surgery; and 3) evaluate the overall impact and benefit of surgery through a comparison of NTM-PD patients who underwent adjuvant lung resection with patients treated exclusively with antibiotics using statistical matching for age, sex, body mass index (BMI), and prognostic factors such as cavity and radiologic features. Furthermore, we investigated the efficacy of measuring the levels of serum IgA antibody against glycopeptidolipid (GPL) core antigen (GPL core antibody) to monitor disease activity and predict recurrence following adjuvant lung resection.

## Methods

### Study design

This retrospective study was performed in accordance with the Declaration of Helsinki and was approved by the institutional research ethics board (TNH-2019033), with a waived requirement for informed consent due to the retrospective nature of the analysis. The medical records of patients of the National Hospital Organization Osaka Toneyama Medical Center between January 2000 and December 2018 were retrospectively reviewed.

### Patient selection

We reviewed the surgery and medical records of our institute, and picked cases of pulmonary resection in patients with NTM-PD who satisfied the diagnostic criteria of American Thoracic Society/Infectious Diseases Society of America (ATS/IDSA) at the timepoint of surgery [3]. The first instance of pulmonary resection for NTM-PD was considered for analysis. After excluding those with surgical lung biopsy of pulmonary nodule for differential diagnosis, we then also excluded NTM-PD patients with resection of NTM nodule, and resection of lung cancer or simple aspergilloma; therefore, only surgical resection for the treatment of NTM-PD in accordance with the 1997 ATS [17] or 2007 ATS/IDSA official statements [3] were included in the analysis. Indications for pulmonary resection in each case were discussed and decided at a weekly multidisciplinary conference of pulmonary physicians and surgeons with consideration of postoperative cardiopulmonary function. Surgical resection was performed in the following cases: 1) NTM-PD refractory to multiple drug therapy; 2) cavitary lesions and/or severe bronchiectasis; or 3) development of complications such as massive hemoptysis [12, 18]. For example, in cases of patients with destroyed lesions such as cavitary lesions and/or severe bronchiectasis, indications for surgery were discussed at the timepoint of diagnosis or 3 to 6 months after multidrug treatment. Although there is no standard definition for the failure of medical therapy, we carefully selected patients who had adequate cardiopulmonary reserve to withstand partial or complete lung resection and who would benefit from resectional surgery, usually after at least 6 months of antibiotic treatment based on expert recommendations [18–20]. Patient records were examined to determine whether they had a known diagnosis of cystic fibrosis or human immunodeficiency virus (HIV) positivity.

### Data collection

We obtained patient data including age, sex, BMI, smoking status, underlying diseases, disease and treatment durations, antimicrobial treatment for NTM-PD, results of bacterial culture and pulmonary function test, and chest computed tomography (CT) findings. A diagnosis of chronic pulmonary aspergillosis was based on European Respiratory Society (ERS) and European Society of Clinical Microbiology and Infectious Diseases (ESCMID) guideline for the management of chronic pulmonary aspergillosis combined with clinical symptoms, radiological findings, positive *Aspergillus* serology, or isolation of *Aspergillus* species from respiratory samples [21]. Preoperative chest CT images of all patients were acquired within a month before surgery and evaluated by two pulmonologists blinded to the clinical data. To assess radiological severity, we used the National Institute for Health and Care Excellence scoring method (NICE score) [22]. The NICE score was calculated as below. Images of CT scans of the right and left lungs were anatomically divided into three zones at levels of the carina and inferior pulmonary vein (total of six zones). In each zone, the lesions were recorded using the following notations: N, nodules (< 10 mm); I, infiltration shadow (an area of opacity > 10 mm); C, cavity; and E, bronchiectasis. Points (from 0 to 4) were assigned to each lesion in each zone as follows: lesions occupying 1–24% of the designated area of each zone = 1 point; 25–49% = 2 points; 50–74% = 3 points; and 75–100% = 4 points. Zero points indicated that no areas were affected. The total score was the sum of the scores of each zone. Postoperative remnant lesions that included small nodules (< 10 mm), infiltration shadow, cavity and bronchiectasis were confirmed by comparing pre- and post-operative CT findings in combination with postoperative visualization of lesions in resected material, as assessed by three pulmonary surgeons and pulmonologists blinded to the clinical data. Discrepancies were resolved through a consensus review. Data on the type of pulmonary resection, postoperative complications and mortality were recorded. Postoperative mortality was defined as death between completion of pulmonary resection and discharge from hospital.

### Sputum examination

A sputum culture examination for acid-fast bacilli (AFB) was performed using the conventional methods of 2% Ogawa egg medium (Japan BCG, Tokyo, Japan) or a mycobacteria growth indicator tube (Japan Becton, Dickinson and Company, Tokyo Japan). Nontuberculous mycobacterial species were identified using the AccuProbe (Gen-Probe Inc., San Diego, CA, USA) or COBAS AMPLICOR (Roche Diagnostic, Tokyo, Japan) systems or by DNA − DNA hybridization assay (Kyokuto Pharmaceutical Industrial, Tokyo, Japan), as previously described [23]. Clarithromycin (CAM) susceptibility was determined by the broth microdilution method (BrothMIC NTM; Kyokuto Pharmaceutical Industrial) [24], and CAM resistance was defined by minimum inhibitory concentrations ≥32 μg/mL.

### Measurement of GPL core IgA antibody levels

Serum levels of GPL core IgA antibody were measured using a commercial enzyme immunoassay (EIA) kit (Tauns Laboratory, Shizuoka, Japan) according to the manufacturer’s instructions [25, 26]; a cutoff value of > 0.5 U/mL was interpreted as positive. Measurement of serum levels of GPL core IgA antibody are commercially available and have been covered by health insurance from 2011 in Japan. Until then, measurements were performed in collaboration with the Tauns Laboratory in our hospital. No subjects were known to be seropositive for HIV, which affects the titer of GPL core IgA antibody because of insufficient immune responses [26, 27]. All patients were followed until their last visit, death, or the end of study period (August 31, 2019).

### Definition of sputum conversion and clinical recurrence

Patient status at the end of follow-up was recorded in terms of living status (deceased or alive), microbiologic or radiologic recurrence, and cause of death, if applicable. Refractory cases were defined as patients who did not achieve negative sputum conversion after surgery. Sputum conversion was defined as > 3 consecutive negative sputum cultures over a period of 3 months. In cases of patients who achieved sputum conversion, clinical recurrence was defined by ≥2 positive sputum cultures or radiologic aggravation, carefully ruling out the acquisition of other pulmonary comorbidities, including infection other than NTM or interstitial pneumonia.

### Non-surgical controls

After 2000, the first adjuvant lung resection for NTM-PD was performed in 2003. All surgically treated patients were aged under 80 years at the time of diagnosis. These factors were considered when selecting the non-surgical controls. Potential non-surgical control participants (i.e. exclusive medical therapy) were retrospectively identified from a group of 569 patients (ICD10 code for NTM-PD and a history of antibiotic treatment) between August 2003 and December 2018. Among them, those who met the following criteria were included: diagnostic criteria that fit the 1997 ATS criteria [17] or the 2007 ATS/IDSA official statement [3], age under 80 years, recorded BMI, and radiologic features of cavity, bilateral shadow or bronchiectasis. Patients who underwent lung resection for NTM-PD were excluded. None of the patients had cystic fibrosis, nor did they test positive for human immunodeficiency virus (HIV). Overall, 216 of the NTM-PD patients were selected as potential control matches for the surgical patients (Supplementary Fig. S1).

### Surgical and non-surgical patient matching

A 1:1 propensity score matching for age, BMI, cavitary lesions, bronchiectasis, and bilateral lesions at the time of diagnosis of NTM-PD was performed for the surgical and non-surgical patient groups using JMP Pro 13 (SAS Institute). To match the nearest neighbor method with the Mahalanobis distance, a caliper of 0.20 and random number seed value of 111 were used.

### Multi-locus sequence typing (MLST) analysis by ONT MinION sequencer

Six NTM isolates, which were obtained from pre- and post- operative culture from 1 refractory patient and 2 recurrent patients, were lysed and extracted DNA using NucleoSpin Microbial DNA (Macherey-Nagel, Duren, Germany) with manufacturer’s instruction. The library was prepared using Ligation Sequencing Kit 1D (SQK-LSK109) (Oxford Nanopore Technologies (ONT), UK) with Native Barcoding Expansion 1–12 (EXP-NBD104) (ONT, UK) according to manufacturer’s instruction. Whole genome sequencing was performed using MinION sequencer with a FLO-MIN106D flowcel (ONT, UK). Raw signal data of fast5 was converted to fastq format by guppy basecaller (v3.5.1) provided by ONT and mapped by minimap2 [28] using a comparable amount of data in pre- and post- operative condition. Data amount for reccurent case 1 (patient no. 35) and refractory case (patient no. 4) were 200 Mb, for reccurent case 2 (patient no.11) was 22 Mb, respectively. MLST analysis was performed using mlstverse [29] and the strain name with the highest scores were compared between pre- and post- operatives.

### Statistical analysis

All statistical analyses were performed using GraphPad Prism version 7 (GraphPad Software, San Diego, CA, USA) and JMP Pro 13 (SAS Institute). Continuous variables are reported as the mean and standard deviation (SD), or median and interquartile range (IQR). Patient groups were compared using the Mann-Whitney *U*-test for continuous variables, and the chi-square test or Fisher’s exact test for categorical variables. Friedman’s matched-pair test with Benjamini, Krieger and Yekutieli’s two-stage correction test was used to compare the anti-GPL core antibody levels before and after surgical treatment. Probability values < 0.05 were regarded as statistically significant.

## Results

### Patient characteristics

During the study period, we detected 43 cases of lung surgery for NTM-PD. Among them, we excluded the resection of NTM nodule (*n =* 3). We further excluded the resection of lung cancer (*n =* 4) or simple aspergilloma (*n =* 1) in NTM-PD patients. Overall, 35 patients (23 females, 65.7%; 12 males, 34.3%) underwent adjuvant pulmonary resection for the treatment of NTM-PD were included in this study (Table 1). None of the patients had cystic fibrosis, nor did they test positive for human immunodeficiency virus (HIV). The median age and BMI of the patients were 58 years (IQR 47–65) and 20.13 kg/m^2^ (IQR 17.93–21.36), respectively, which were comparable to previous reports [11, 16, 18].

**Table 1:** Baseline characteristics of 35 patients surgically treated for NTM-PD

Characteristics	No. (%) or median (IQR)
Sex, female	23 (65.7)
Age, median (IQR)	58 (47–65)
Body mass index, mean (SEM)	20.13 (17.93–21.36)
Smoking status, never/former/current	28 (80)/4 (11.4)/3 (8.6)
Underlying disease/history	
Prior TB	5 (14.3)
COPD	1 (2.9)
Diabetes mellitus	2 (5.7)
Lung resection	2 (5.7)
Species/group	
*Mycobacterium avium*	18 (51.4)
*M. intracellulare*	10 (28.6)
*M. kansasii*	1 (2.9)
*M. abscessus*	5 (14.3)
Undetermined	1 (2.9)
CAM-resistant strains	8 (22.9)
Chronic pulmonary aspergillosis	3 (8.6)
CT pattern	
Fibrocavitary	17 (48.6)
Nodular bronchiectasis	12 (34.3)
Unclassifiable	3 (8.6)
CT findings	
cavitation	32 (91.4)
bronchiectasis	25 (71.4)
Pre- and postsurgical treatment^a^	
No treatment	3 (8.6)
On treatment	32 (91.4)
CAM-included regimen ≥3 drugs	29 (82.9)
CAM-included regimen with 2 drugs	1 (2.9)
CAM monotherapy	0 (0.0)
Non-CAM-included regimen	2 (5.7)
Treatment duration (months), median (IQR)	
Pre-surgical	5.5 (2–34)
Post-surgical	12 (3–20)
Preoperative sputum examinations	
Positive AFB stain	10 (28.6)
Positive AFB culture	16 (45.7)
Indications of surgery	
Poor response to medical therapy	7 (20.0)
Remnant destroyed lesions	23 (65.7)
Massive hemoptysis	5 (14.3)
Preoperative PFT, median (IQR)	
% FEV1, %	87 (73.9–101.6)
% FVC, %	95.4 (80.2–106.2)
Postoperative PFT, median (IQR)	
% FEV1, %	72.1 (61.63–95.25)
% FVC, %	70.5 (56.25–92.15)

^a^The main therapy was evaluated during the 6 months of preoperation and postoperation

Although most (28; 80%) patients had never smoked, four (11.4%) and three (8.6%) patients were former and current smokers, respectively. The main underlying diseases included old pulmonary tuberculosis (5; 14.3%), chronic obstructive pulmonary disease (1; 2.9%), and diabetes mellitus (2; 5.7%). Of the bacterial species that were identified, *M. avium* (18; 51.4%) was the most frequent, followed by *M. intracellulare* (10; 28.6%), *M. abscessus* (5; 14.3%), *M. kansasii* (1; 2.9%), and other species (1; 2.9%). Among 28 patients with MAC-PD, CAM resistance was found in eight patients. Most (30; 85.7%) patients received CAM including combination antimicrobial therapy before and after surgery for NTM-PD. No patients received monotherapy with CAM. Two (5.7%) patients received a non-CAM regimen because of CAM resistance, although another six patients who developed CAM resistance continued the CAM containing regimen in the hope of immunomodulatory effects. Three (8.6%) patients did not receive any antimicrobial therapy. Among the three patients not exposed to antibiotics, two refused antibiotic therapy and one had intolerance of antimicrobials. The median treatment duration of NTM-PD to pulmonary resection was 5.5 months (IQR 2–34). The results of preoperative pulmonary function were mostly within normal ranges: % predicted forced expiratory volume in 1 s and forced vital capacity of 87.0 and 95.4% (IQR 73.9–101.6 and 80.2–106.2), respectively.

### Characteristics of pulmonary resection and postoperative complications

Indications for surgery included poor response to drug therapy (7; 20.0%), destructed lesions such as cavitary lesions and/or severe bronchiectasis (23; 65.7%), hemoptysis (5; 14.3%), or a combination of these conditions (Table 1). No patients died during surgery. After pulmonary resection, 22 (62.9%) patients exhibited remnant lesions, no patients exhibited remnant cavitary lesions, eight (22.9%) had bronchiectasis, and 11 (31.4%) exhibited contralateral nodules or infiltrations.

Pulmonary resection included pneumonectomy (4; 11.4%), lobectomy with/without segmentectomy or wedge resection (26; 74.3%), bilobectomy with/without segmentectomy or wedge resection (3; 8.6%), or segmentectomy with/without wedge resection (2; 5.7%) (Table 2). Pulmonary resection was performed by thoracotomy in 17 patients and by thoracoscopy in 18 patients. The median operation time was 233.5 min (IQR 188–339.8) for thoracotomy and 177 min (IQR 143.3–210.8) for thoracoscopy. The median volume of blood loss was 135.5 mL (IQR 85–599.8) in thoracotomy and 50 mL (IQR 2.5–151.8) in thoracoscopy. Postoperative complications occurred in seven (20%) patients, and their rate of occurrence was higher after pneumonectomy (3/4; 75%) compared with other types of pulmonary resection (5/31; 16.1%). Pyothorax (2; 5.7%) was the most frequent postoperative complication. No patients died before being discharged from the hospital.

**Table 2:** Types and numbers of lung resection and postoperative complications in the study patients

Type of resection	No. (%) lung resections, *n* = 35	Complications
Pneumonectomy	4 (11.4)	Respiratory failure (1), Pleuritis (1), Pyothorax (1)
Bilobectomy	2 (5.7)	
Bilobectomy + segmentectomy	1 (2.9)	
Lobectomy	17 (48.6)	Hematoma (1), Pyothorax (2)
Lobectomy + segmentectomy	9 (25.7)	Bronchopleural fistula and Subcutaneous emphysema (1)
Segmentectomy	2 (5.7)	
**Surgical approach, no. (%)**	**Thoracotomy 17 (48.6)**	**Thoracoscopy 18 (51.4)**
Median operation time, min (IQR)	212 (174.5–336.5)	177 (143.3–210.8)
Median intraoperative blood loss, mL (IQR)	161 (90–535.5)	50 (2.5–151.8)

### Incidence and predictive factors of refractory and recurrent cases after pulmonary resection

**Fig. 1:**
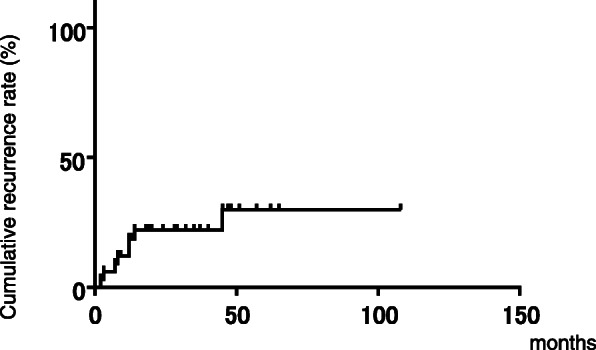
Cumulative recurrence rate of surgically treated NTM-PD patients. The rates at 12, 36 and 60 months after surgery were 18.63, 22.16, and 29.95%, respectively

During the observation period, 33 patients (94.3%) achieved sputum conversion (Table 3). Among these patients, the median duration of antibiotic therapy was 12.0 months and eight patients experienced clinical recurrence (5 bacterial; 3 radiologic). The cumulative recurrence rates at 12, 36 and 60 months after surgery were 18.63, 22.16 and 29.95%, respectively (Fig. 1). Next, we investigated predictive factors of refractory and recurrent cases after pulmonary resection. Differences among the different species causing NTM-PD were not statistically significant. Preoperative antibiotic treatments were significantly longer in refractory and recurrent cases (*p =* 0.0464), whereas a significantly higher proportion of the non-recurrent group were administered combination antibiotic treatments that included CAM and consisted of more than three drugs. Univariate analysis revealed that the existence of postsurgical remnant bronchiectasis (odds ratio [OR] 17.25; *p =* 0.0028), contralateral nodule or infiltration (OR 29.33; *p =* 0.0003), and positive AFB stain/culture (OR 17.11/8.5; *p =* 0.0016/0.0218) in preoperative sputum examinations were predictors of refractory/recurrent cases (Table 4).

**Table 3:** Long-term outcomes of surgical NTM-PD patients

Outcomes	
Culture conversion, n (%)	33 (94.3)
Median duration of post-surgical antibiotics, months (IQR)	12 (3–20)
Mortality, n (%)	1 (2.9)

**Table 4:** Risk factors for refractory/recurrent cases after pulmonary resection for NTM-PD

Characteristic	Refractory/ recurrent cases, *n* = 10	Censored cases, *n* = 25	*P* value	Odds ratio	95% CI
Female sex n	9 (90.0)	14 (14 (56.0)	0.1126		
Age, median (IQR)	67.5 (54–72)	58.5 (49–63)	0.282		
Body mass index, median (IQR)	18.85 (17.21–21.76)	20.26 (18.25–21.36)	0.4804		
Underlying disease/history					
Prior TB	1 (10.0)	4 (16.0)	> 0.9999		
COPD	0 (0.0)	1 (4.0)	> 0.9999		
Diabetes mellitus	0 (0.0)	2 (8.0)	> 0.9999		
Lung resection	0 (0.0)	1 (4.0)	> 0.9999		
Species/group					
*Mycobacterium avium*	8 (80.0)	10 (40.0)	0.0599		
*M. intracellulare*	1 (10.0)	9 (36.0)	0.2181		
*M. kansasii*	0 (0.0)	1 (4.0)	> 0.9999		
*M. abscessus*	0 (0.0)	5 (20.0)	0.2915		
Undetermined	1 (10.0)	0 (0.0)	0.2857		
CAM-resistant strains	2 (20.0)	6 (24.0)	> 0.9999		
Chronic pulmonary aspergillosis	1 (10.0)	2 (8.0)	> 0.9999		
CT pattern					
Fibrocavitary	3 (30.0)	14 (56.0)	0.2642		
Nodular bronchiectasis	4 (40.0)	11 (44.0)	> 0.9999		
Unclassifiable	3 (30.0)	0 (0.0)	0.0183		2.496–∞
Median duration of pre-surgical treatment, months (IQR)	24.5 (6.25–65.75)	3 (1.25–21.25)	0.0464		
Antibiotic treatment – N (%)					
No treatment	1 (10.0)	2 (8.0)	> 0.9999		
On treatment	9 (90.0)	23 (92.0)			
CAM-included regimen ≥3 drugs	6 (60.0)	23 (92.0)	0.0169		
CAM-included regimen 2 drugs	1 (10.0)	0 (0.0)	0.7094		
CAM monotherapy	0 (0.0)	0 (0.0)	> 0.9999		
Non-CAM-included regimen	2 (20.0)	0 (0.0)	0.2581		
Pre-surgical radiological score (NICE score based on CT findings)	9 (5.5–14.5)	5 (3–9)	0.0496		
Post-surgical legion					
Remnant lesion after surgery	9 (90.0)	13 (52.0)	0.0548		
Remnant bronchiectasis	6 (60.0)	2 (8.0)	0.0028	17.25	2.38–94.81
Remnant contralateral nodule or infiltration	8 (80.0)	3 (12.0)	0.0003	29.33	4.165–160
Preoperative sputum examinations					
Positive AFB stain	7 (70.0)	3 (12.0)	0.0016	17.11	2.814–79.56
Positive AFB culture	8 (80.0)	8 (32.0)	0.0218	8.5	1.666–43.85
Indications of surgery					
Poor response to medical therapy	3 (30.0)	4 (16.0)	0.3811		
Remnant destroyed lesions	5 (50.0)	18 (72.0)	0.2577		
Massive hemoptysis	2 (20.0)	3 (12.0)	0.6103		

### Survival after pulmonary resection

The median duration of postoperative follow-up of the 35 patients was 28.0 months (IQR 13–48 months). There were no postoperative deaths. At the end of follow-up, only one patient had died of respiratory failure, at 71 months after surgery (Table 3).

### Comparison of characteristics and outcomes of the surgical and non-surgical groups

**Fig. 2:**
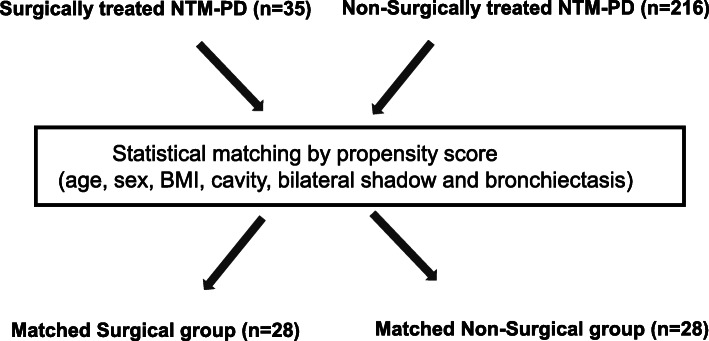
Work flow of the statistical matching of surgically and non-surgically treated NTM-PD patients

Of 35 surgically treated patients and 216 non-surgically treated patients, 1:1 statistical matching with propensity score was performed. Seven surgically treated patients were unmatched with non-surgical patients. Therefore, 28 surgically treated and non-surgically treated patients were matched (Fig. 2). Characteristics and outcomes of the statistically matched, surgically treated, and non-surgical control patients (*n =* 28 each) are presented in Table 5, and Supplementary Table S1. Baseline prognostic factors including BMI and cavitary lesions [16], radiologic features, and observation time were comparable between the two groups. In univariate logistic regression analysis (Table 5), sustained negative culture conversion was significantly higher in the surgical group compared with the non-surgical group (82.2% vs 50.0%, *p =* 0.0438). Although not statistically significant, one patient died in the surgical group and four patients died in the non-surgical group (*p =* 0.3516).

**Table 5:** Analysis of impact of adjuvant surgery on NTM-PD patients

Outcome	Matched surgical group, *n* = 28	Matched non-surgical group, *n* = 28	*P* value
Median no. of hospitalizations (IQR)	2 (1.25–4.75)	2 (1–3.75)	0.3868
AFB culture-negative at the end of observation, no. (%)	23 (82.2)	14 (50.0)	0.0438
Mortality, no. (%)	1 (3.6)	4 (14.3)	0.3516

### Change in GPL core antibody levels with surgery

**Fig. 3:**
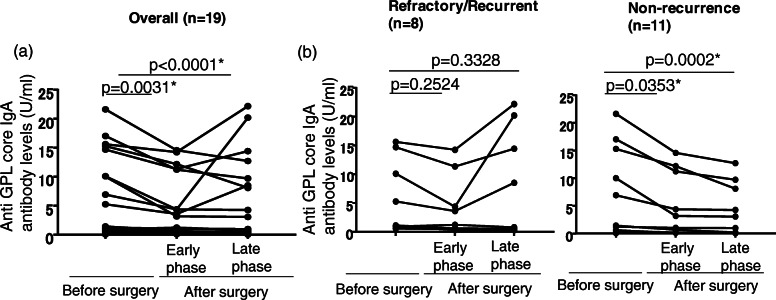
Levels of GPL core serum IgA antibody before and after surgery. Overall levels (**a**) and (**b**) individual patient levels of GPL core serum IgA antibody in the refractory/recurrent and recurrence-free subgroups. All results are expressed as individual data before and after surgery. *P* values were calculated using the Friedman matched-pair test with Benjamini, Krieger and Yekutieli’s two-stage correction

Antibody levels were obtained in 19 MAC-PD patients before surgery and at two timepoints after surgery (early phase, 2 (IQR 1–3) months; late phase, 13 (IQR 12–16) months) (Fig. 3a). Sixteen of these patients were positive (> 0.5 U/mL) for GPL core antibody levels before surgery. The median GPL core serum antibody levels (*n =* 19) were significantly different before and after surgery: 1.43 U/mL (IQR 0.59–14.7) before surgery; 1.22 U/mL (IQR 0.33–11.23;) in the early phase (*p =* 0.031 vs before surgery); and 0.98 U/mL (IQR 0.19–9.72) in the late phase after surgery (*p <* 0.0001 vs before surgery).

In the non-recurrence subgroup (*n =* 11), the median GPL core serum antibody levels were also significantly different before and after surgery: 1.43 U/mL (IQR 0.46–15.3) before surgery; 1.04 U/mL (IQR 0.2–11.23) in the early phase (*p =* 0.0347 vs before surgery); and 0.98 U/mL (IQR 0.09–8.1) in the late phase after surgery (*p <* 0.0001 vs before surgery) (Fig. 3b). In contrast, in the refractory/recurrent subgroup (*n =* 8), the differences in median GPL core serum antibody levels did not reach statistical significance: 3.175 U/mL (IQR 0.8375 to 13.55) before surgery; 2.405 U/mL (IQR 0.4725–9.605) in the early phase (*p =* 0.2524 vs before surgery); and 4.65 U/mL (IQR 0.44–18.76) in the late phase after surgery (*p =* 0.3328 vs before surgery) (Fig. 3b). In the non-recurrence subgroup (*n =* 11), the mean (SD) % reductions in GPL core serum antibody level following surgery were 37.11% (24.61) in the early phase and 53.39% (23.63) in the late phase. In the refractory/recurrent subgroup (*n =* 8), the mean (SD) % reductions in GPL core serum antibody level after surgery were 26.2% (31.12) in the early phase and − 6.347% (56.82) in the late phase (Supplimentary Table S2).

### Change in GPL core antibody levels with combination antibiotic treatment

We also measured the GPL core antibody levels in non-surgical control patients. Among 182 MAC-PD patients, 62 patients had anti-GPL core IgA antibodies measured before antibiotic therapy and at two timepoints after antibiotic therapy (early phase, 2 (IQR 1–3) months; late phase, 12 (IQR 11–13) months. Patient characteristics and treatment regimens are shown in Supplementary Table S3. We confirmed the significant reduction of GPL core IgA antibody following combinational antibiotic therapy in the overall population (*n =* 62) (Supplementary Fig. S2). In the sub-group analysis, we observed a significant reduction in the culture conversion group both in the early phase and late phase (*n =* 40). Furthermore, the reduction of GPL core IgA antibody between the early phase and late phase was statistically significant in the non-recurrence group (*n =* 23, *p =* 0.0239) and non-significant in the recurrence group (*n =* 17, *p >* 0.9999). However, in the non-conversion group (*n =* 22), the reduction was not significant in the early phase (*p >* 0.9999) or late phase (*p >* 0.9999).

## Discussion

This study evaluated the treatment outcomes and prognostic factors in 35 patients who underwent adjunctive pulmonary resections for NTM-PD. Approximately 90% of the included patients achieved sputum conversion, while the rates of postoperative complications after pneumonectomy and other types of pulmonary resection were 75 and 16.1%, respectively. These results are in line with those of previous studies involving patients treated by pneumonectomy [14, 30] and other types of pulmonary resection [10–12, 16]. In most previous reports, refractory cases—those who did not achieve sputum conversion after lung surgery—were excluded from the analysis of prognostic factors, which may have led to the underestimation of factors that contributed to treatment failure. Therefore, we propose that it is just as important to analyze the factors that disrupt or delay sputum conversion as it is to analyze those that affect recurrence after conversion. Furthermore, sputum examination cannot detect every recurrent case; therefore, careful radiologic follow-up combined with serologic and physical assessment is necessary for the early detection of recurrence. Thus, we expanded our analysis to include radiologic assessment for the exploration of predictive factors for refractory and clinical recurrence following adjuvant lung resection, which are extremely important. Analysis of prognostic factors for refractory/recurrent cases produced three significant findings. Positive preoperative AFB staining or culture predicts high rates of relapse/recurrence. Remnant bronchiectasis and contralateral nodule/infiltration augments the rate of refractory/recurrent cases. This finding was consistent with a previous report that NTM-PD patients with bronchiectatic disease frequently relapsed after treatment completion [6]. The reason for this is still unclear, it might be that postoperative remnant bronchiectatic lesions serve as a potential reservoir that triggers relapse/recurrence. Previous study suggested that reinfection was reported to be the major cause of recurrence after antibiotic treatment [23]. In this study of surgical patients, pre- and post- operative bacterial culture isolates were available in one refractory patient and two recurrent patients among seven bacteriologically proven refractory/recurrent patients. We performed MLST analysis using whole genome information obtained by next-generation sequencing. The resulting MLST scores indicates isolates were identical in pre- and post- operatives in all patients, respectively. Accordingly, in cases of NTM-PD with positive AFB staining or culture that are refractory to multiple drug therapy, clinicians should consider the notable risk of relapse/recurrence in patients with remnant bronchiectasis and contralateral nodule/infiltration after adjuvant lung surgery. Additionally, CAM resistance and coinfection with *Aspergillus* are known risk factors leading to a poor outcome of combination chemotherapy [24, 31, 32]; however, neither was related to refractory/recurrent status after surgery in this study. In patients with possible *Aspergillus* coinfection, we attempted to surgically remove all cavitary and bronchiectatic lesions that might be infected by *Aspergillus*, and antifungal treatment was given as necessary. In this study, three patients were diagnosed as chronic pulmonary aspergillosis. Among them, *Aspergillus fumigatus* was isolated in two patients, and one patient was diagnosed by histologically confirmed mycelium, positive aspergillus antigen and β-D-glucan. Although fungal culture was negative in this patient, the attending physician assumed that fungal culture negativity was associated with effective anti-fungal therapy. Indeed, proper diagnosis and treatment of complications in NTM-PD are essential for the comprehensive management of this disease. Accordingly, we have shown favorable outcomes in NTM-PD patients who underwent lung resection along with multidrug treatment. However, indications for lung resection are not well established, and baseline characteristics in patients who are considered for surgery may differ from those who are not, so a general comparison may be substantially biased. To solve these problems, we employed a strict statistical method to perform unbiased matching. Even in this setting, adjuvant lung resection was associated with more favorable outcomes. In our study, of surgically treated patients, CAM resistance was detected in eight (28.6% of MAC-PD) patients, and 10 (28.6%) patients were treated with intensive combination therapy for more than 2 years. The patients who underwent lung resection because of the presence of refractory and progressive disease despite long-term medical management may have reflected a higher rate of more diffuse or aggressive infections than has been included in previous reports [16, 33]. Furthermore, remnant lesions were detected in 22 (66.3%) patients and 10 (28.6%) patients that had bilaterally distributed disease. These findings are also associated with diffuse or aggressive infection. Despite having progressive disease, surgically treated patients achieved a significantly higher rate of negative sputum conversion with a longer duration, which would be expected to lead to a better prognostic outcome with appropriate lifelong follow-up. In addition, mortality appeared to be lower in the surgical group than in the non-surgical group. Prospective, large-scale studies are needed to validate further the impact of adjuvant surgery on survival benefit.

In this study, we attempted to monitor disease activity and predict the recurrence of disease by measuring GPL core IgA antibody levels after adjuvant lung resection. We developed a serodiagnosis kit that measures the serum levels of IgA antibodies against the GPL core, which has good diagnostic accuracy for MAC disease [25–27], and which has been commercially available in Japan since 2011. To date, the significance of GPL core IgA antibody level measurement for monitoring recurrence in patients with MAC-PD who have undergone adjuvant lung surgery was poorly understood. By obtaining EIA data before and after surgery, we were able to demonstrate that the level of GPL core antibodies reflects disease activity. Based on the results of this analysis, a > 40% reduction in the early phase, and continuous reduction until the late phase, would be a good indicator of a favorable outcome, whereas a < 20% reduction in antibodies in the early phase, and an increase in the late phase, would be an indicator of an unfavorable outcome. Of note, three patients with negative GPL core antibody levels before surgery, and two patients who attained negative GPL core antibody levels after surgery, achieved culture conversion and remained recurrence free for a median follow-up time of 31 months (IQR 28–45). These results strongly suggest that the level of GPL core antibodies can be used to monitor disease activity after adjuvant lung surgery. This finding is particularly important because there is currently no accurate serologic marker for the recurrence of MAC-PD following adjuvant lung surgery. Furthermore, to confirm the value of serial measurements of GPL core antibody levels to monitor disease activity in individual MAC-PD patients, we measured the GPL core antibody levels in non-surgical control patients and showed significant correlation with disease activity. These findings are in accord with the results of our previous prospective study [34]. Taken together, the serial measurements of GPL core antibody levels are a promising marker for monitoring disease activity in MAC-PD patients.

This study had some limitations. First, because of the retrospective study design, we could not exclude potential confounding factors such as microbiologic and other laboratory data. Second, this study included patients treated with a shorter duration of antimicrobial therapy than experts now recommend, which might have affected the clinical outcome following surgery. Although the optimal duration of antimicrobial therapy after pulmonary resection is unclear, experts have stated that patients with NTM-PD after adjuvant surgery should be treated for > 12 months [12, 20]. Because we included patients from the year 2003, when the optimal treatment duration was poorly understood, some patients received a shorter treatment than what is now recommended. Third, surgical patients were selected from only one referral center with experience in surgical management of NTM-PD, which might have led to beneficial selection bias. However, it is important to note that the preoperative disease severity of most patients in this study was higher than that in previous reports, including the proportion of patients with both cavitary and bronchiectatic lesions, bilateral lesions or severe radiologic scores, making this study ideal for the exploration of factors that affect the clinical outcome. In most previous reports of surgical NTM-PD, the surgical patients were highly selected, which might have acted as a form of selection bias leading to confounding results. Finally, we did not evaluate the factors affecting decision-making by pulmonary physicians and surgeons with regard to the timing and type of pulmonary resection and antimicrobial therapy.

We were also able to identify significant factors associated with refractory/recurrent cases following pulmonary resection after long-term follow-up. Given that these factors are associated with advanced NTM-PD, surgery should be considered earlier, especially in cases of patients with drug-resistant NTM-PD. Moreover, surgical resection with antimicrobial therapy has been demonstrated to provide better clinical outcomes in patients with *M. abscessus* complex infections and CAM-resistant MAC-PD [4, 12, 19, 24, 35, 36]. Being a monocentric study, even though from a reference center, our numbers are inevitably small and statistics are weak despite the matching with similar non-surgical patients. To determine the optimal timing of surgery and identify criteria for the selection of patients who might benefit from surgery, a larger international multicentric study could provide stronger indication for adjuvant lung resection of NTM-PD.

## Conclusions

Adjuvant pulmonary resection combined with chemotherapy was associated with favorable treatment outcomes with an acceptable complication rate. Selected NTM-PD patients seem to have benefited from surgery even if they had a poor response to drug therapy or had negative prognostic factors such as low BMI, cavitation, unresectable bronchiectasis, and contralateral nodules/lesions. Clinicians should not hesitate to carefully select patients with NTM-PD who can benefit from adjuvant surgical resection. The optimal selection criteria, timing, and duration of postoperative medical therapy require further evaluation.

## Supplementary information

**Additional file 1: Figure S1.** Work flow of the identification non-surgically treated NTM-PD patients. **Figure S2.** Levels of GPL core serum IgA antibody before and after combinational antibiotic treatment. Overall levels (a) and (b) individual patient levels of GPL core serum IgA antibody in the non-conversion, culture conversion, non-recurrence, and culture conversion and non-recurrence subgroups. All results are expressed as individual data before and after surgery. *P* values were calculated using the Friedman matched-pair test with Benjamini, Krieger and Yekutieli’s two-stage correction. **Figure S3.** MLST score obtained by mlstverse. Isolates from two reccurent patients [26] and one refractory patient (c) were sequenced and analyzed. Identified species were showed with strain name with the highest score. (a) reccurent case 1 (patient no. 35), (b) reccurent case 2 (patient no.11), (c) refractory case, (patient no. 4). **Table S1**. Baseline characteristics of matched paires. **Table S2**. Analysis of reduction levels of GPL core serum antibody after surgery.

## Data Availability

The datasets supporting the conclusions of this article are included within the article and its additional supplemental files.

## Abbreviations

AFB: Acid-fast bacilli
ATS: American Thoracic Society
BMI: Body mass index
CAM: Clarithromycin
CT: Computed tomography
EIA: Enzyme immunoassay
GPL: Glycopeptidolipid
HIV: Human immunodeficiency virus
IDSA: Infectious Diseases Society of America
IQR: Interquartile range
MAC: *Mycobacterium avium* complex
MAC-PD: MAC pulmonary disease
NTM-PD: Nontuberculous mycobacterial pulmonary disease
OR: Odds ratio
SD: Standard deviation
