# Supplementary material for: The impact of adjuvant surgical treatment of nontuberculous mycobacterial pulmonary disease on prognosis and outcome
Source: Respir Res. 2020 Jun 16;21:153. doi: 10.1186/s12931-020-01420-1 (PMC7298848; doi:10.1186/s12931-020-01420-1)

Supplimentary items for

The Impact of adjuvant surgical treatment of Nontuberculous Mycobacterium Pulmonary disease on prognosis and outcome

Supplimentary Table.1 Baseline characteristics of matched paires

Characteristics	Matched Surgical group (n=28)	Matched Non-surgical group (n=28)	P value
Sex, female	18 (64.3)	19 (67.9)	>0.9999
Age, median (IQR)	58 (50.5 - 63)	60.5 (52 – 66.25)	0.699
Body mass index, median (IQR)	19.84 (17.74 – 20.95)	18.79 (16.57 – 22.07)	0.476
Underlying disease			
・COPD	1 (3.6)	0 (0.0)	>0.9999
・Diabetes mellitus	1 (3.6)	3 (10.7)	0.611
CT findings			
・Cavity	26 (92.9)	25 (89.3)	>0.9999
・Bilateral shadow	9 (32.1)	13 (46.4)	0.4121
・Bronchiectasis	15 (53.6)	15 (53.6)	>0.9999
Species/group			
・M.avium	13 (46.4)	12 (42.9)	>0.9999
・M.intracellulare	9 (32.1)	10 (35.7)	>0.9999
・M.kansasii	0 (0.0)	2 (7.1)	0.4909
・M.abscessus	5 (17.9)	3 (10.7)	0.7049
・M.xenopi	0 (0.0)	1 (3.6)	>0.9999
Observation time (months), median (IQR)	64.6 (35.3 - 102.0)	60.9 (45.2 – 84.1)	0.6084

Supplimentary Table 2 Analysis of reduction levels of GPL core serum antibody after surgery.

	**Refractory/Recurrent (n=8)**		**Non-recurrence (n=11)**	
	Early phase after surgery	Late phase after surgery	Early phase after surgery	Late phase after surgery
% reduction after surgery, mean (S.D.)	26.2 (31.12)	-6.347 (56.82)	37.11 (24.61)	53.39 (23.63)

Supplimentary Table.3 Baseline characteristics of MAC-PD patients measured anti GPL core IgA antibody before and at two time points after chemotherapy (early phase, 1-4 months; late phase, 10-16 months.).

**Characteristics**	**GPL core IgA antibody monitored MAC-PD (n=62)**
Sex, female	42 (67.7)
Age, median (IQR)	66.5 (59 - 71)
Body mass index, mean (S.E.M)	17.06 (15.96 - 20.65)
Species/group	
・M.avium	40 (64.5)
・M.intracellulare	10 (35.5)
CT pattern	
・Fibrocacitary	17 (48.6)
・Nodular bronchiectasis	12 (34.3)
・Unclassifiable	3(8.6)
CT findings	
・cavitation	39 (62.9)
・Bilateral shadow	34 (54.8)
・bronchiectasis	22 (35.5)
Antibiotic therapy	
CAM-included regimen ≧3 drugs	54 (87.1)
CAM-included regimen with 2 drugs	7 (11.3)
CAM monotherapy	0 (0.0)
Non-CAM-included regimen	1 (1.6)

Supplimentary Figure.1

Initial screening

ICD10 code for NTM-PD
and
History of antibiotic treatment

→ 569 candidates →

Inclusion criteria
Met ATS criteria for NTM-PD,
Cavity or bilateral shadow or bronchiectasis,
Age<80years, known BMI
.
Exclusion criteria
Underwent adjuvant lung resection for NTM-PD
Known diagnosis of cystic fibrosis or HIV positivity

↓

Identified

216 NTM-PD patients treated exclusively with antibiotics

**Characteristics**	**Non-Surgically treated NTM-PD (n=216)**
Sex, female	154 (71.3)
Age, median (IQR)	67 (59.25 - 71)
Body mass index, median (IQR)	18.21 (16.19 – 19.84)
CT findings	
・Cavity	115 (53.2)
・Bilateral shadow	111 (51.4)
・Bronchiectasis	96 (44.4)
Species/group	
・M.avium	122 (56.5)
・M.intracellulare	60 (27.8)
・M.kansasii	15 (6.9)
・M.abscessus	18 (8.3)
・M.xenopi	1 (0.5)

Supplimentary Figure.2

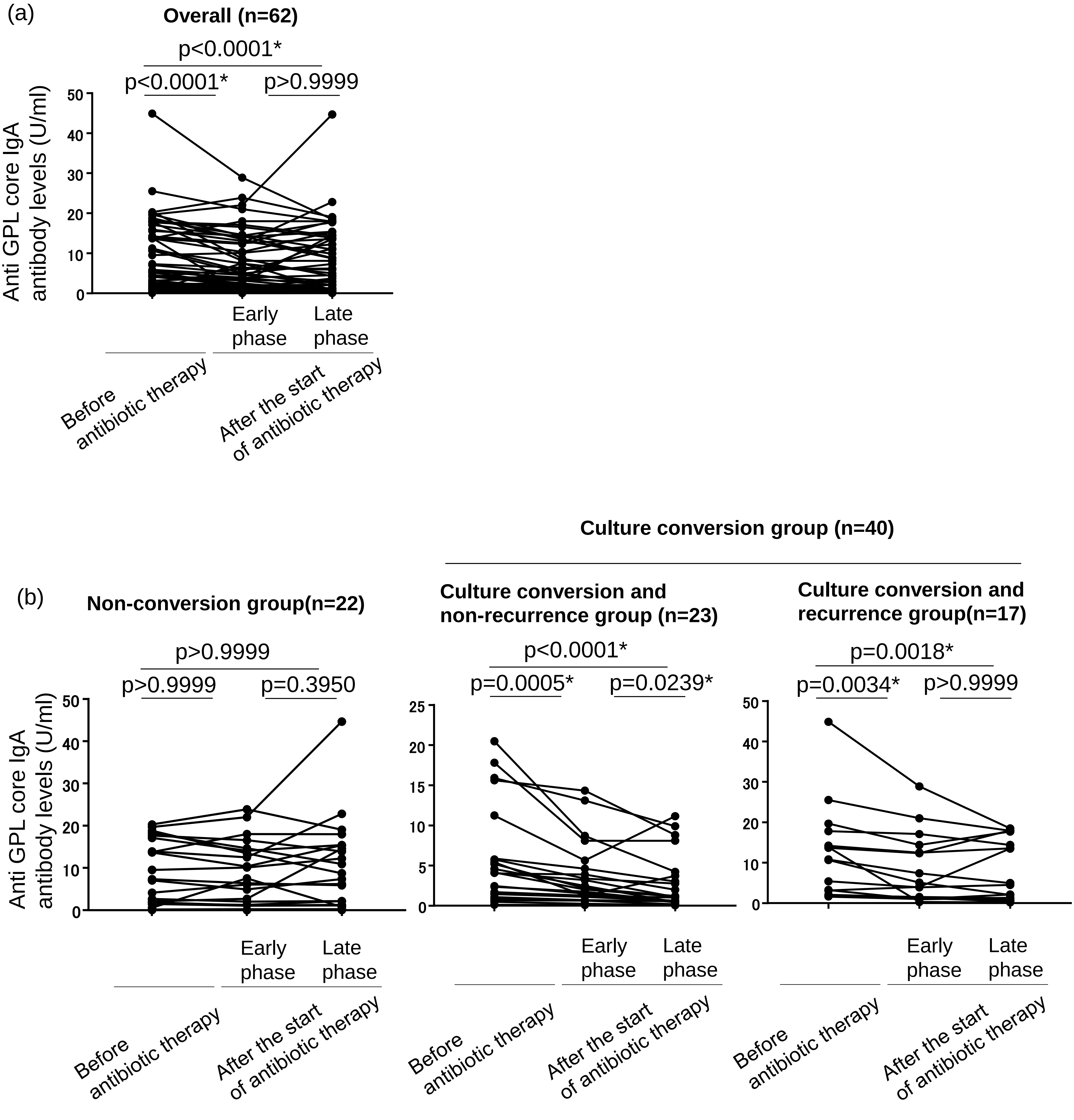

Supplimentary Figure.3

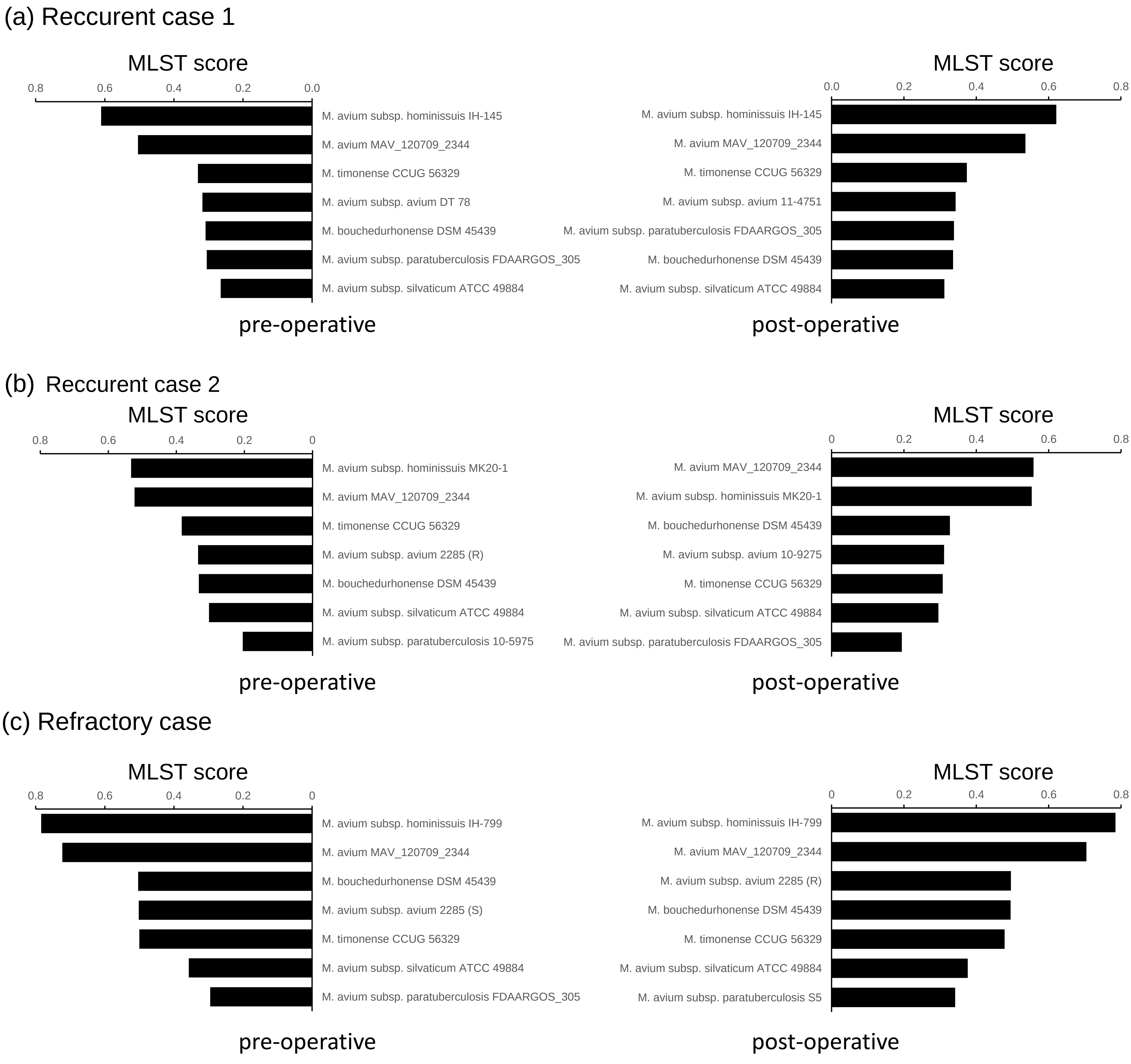

Supplement: Supplementary file 1 — Additional file 1: Figure S1. Work flow of the identification non-surgically treated NTM-PD patients. Figure S2. Levels of GPL core serum IgA antibody before and after combinational antibiotic treatment. Overall levels (a) and (b) individual patient levels of GPL core serum IgA antibody in the non-conversion, culture conversion, non-recurrence, and culture conversion and non-recurrence subgroups. All results are expressed as individual data before and after surgery. P values were calculated using the Friedman matched-pair test with Benjamini, Krieger and Yekutieli’s two-stage correction. Figure S3. MLST score obtained by mlstverse. Isolates from two reccurent patients [26] and one refractory patient (c) were sequenced and analyzed. Identified species were showed with strain name with the highest score. (a) reccurent case 1 (patient no. 35), (b) reccurent case 2 (patient no.11), (c) refractory case, (patient no. 4). Table S1. Baseline characteristics of matched paires. Table S2. Analysis of reduction levels of GPL core serum antibody after surgery. [file 12931_2020_1420_MOESM1_ESM.pdf]
